# Interactions Between Epidermal Growth Factor–Containing Fibulin-Like Extracellular Matrix Protein 1 and Tissue Inhibitor of Metalloproteinases-3 and Relevance to Age-Related Macular Degeneration

**DOI:** 10.1016/j.xops.2026.101287

**Published:** 2026-06-16

**Authors:** Clara Ehrenzeller, Robert E. MacLaren

**Affiliations:** 1 Nuffield Laboratory of Ophthalmology, Nuffield Department of Clinical Neurosciences, University of Oxford, Oxford, UK; 2 Oxford Eye Hospital, University of Oxford, Oxford, UK

**Keywords:** AMD, Bruch's membrane, Doyne Honeycomb Retinal Dystrophy, EFEMP1, Extracellular matrix, TIMP3

## Abstract

**Clinical Relevance:**

Age-related macular degeneration (AMD) remains the leading cause of irreversible blindness in Western populations, with no approved therapies for the dry form characterized by drusen accumulation and retinal pigment epithelium atrophy. This review examines extracellular matrix alterations in Bruch's membrane by comparing healthy aging, AMD pathogenesis, and Doyne Honeycomb Retinal Dystrophy—a monogenic disorder that serves as a surrogate model for AMD due to similar phenotypic manifestations.

**Methods:**

We focus on the critical interactions between epidermal growth factor–containing fibulin-like extracellular matrix protein 1 (EFEMP1), matrix metalloproteinases (MMPs), tissue inhibitor of metalloproteinases-3, and complement factors. We explore their roles in extracellular matrix homeostasis disruption. In healthy aging, oxidative stress and inefficient waste removal drive gradual matrix remodeling and low-grade inflammation. Age-related macular degeneration results from polygenic risk variants in complement and extracellular matrix genes, combined with environmental stressors, leading to accelerated matrix dysfunction and chronic complement activation. In Doyne Honeycomb Retinal Dystrophy, the EFEMP1 R345W mutation causes rapid disease progression through impaired protein secretion, abnormal matrix accumulation, and complement dysregulation.

**Results:**

Our analysis reveals that EFEMP1–tissue inhibitor of metalloproteinases-3 complexation may represent a critical threshold in drusen formation across both conditions, AMD and Doyne Honeycomb Retinal Dystrophy.

**Conclusion:**

While AMD pathogenesis unfolds over decades through cumulative insults, the EFEMP1 mutation compresses similar pathological changes into 30 to 40 years, suggesting this mutation acts as a major hazard for matrix homeostasis disruption. Understanding these shared mechanisms provides insights into therapeutic targets, including complement inhibition, MMP modulation, and EFEMP1-directed interventions. We propose continued investigation of Doyne Honeycomb Retinal Dystrophy as a valuable model for identifying AMD treatments.

**Financial Disclosures:**

Proprietary or commercial disclosure may be found in the Footnotes and Disclosures at the end of this article.

Age-related macular degeneration (AMD) is the leading cause of irreversible blindness in the Western world.^1^ Despite its prevalence, no approved therapy exists for the more common dry form of the disease, which is characterized by the accumulation of drusen and progressive atrophy of the retinal pigment epithelium (RPE).^1^ Because AMD is multifactorial and strongly influenced by aging, one strategy to better understand its pathogenesis is to study monogenic diseases with similar phenotypic manifestations.^2^

Doyne Honeycomb Retinal Dystrophy, also known as Malattia Leventinese, represents such a surrogate model,^2^^,^^3^ as patients develop drusen-like deposits and structural changes that closely resemble those observed in AMD.^3^

This review aims to clarify the overlap between these disease entities by focusing on extracellular matrix processes within Bruch’s membrane. We compare extracellular matrix homeostasis in healthy young and aged individuals with the one in patients affected by AMD and Doyne Honeycomb Retinal Dystrophy, with the goal of identifying shared mechanisms as well as disease-specific differences.

## Healthy Extracellular Matrix Homeostasis

Bruch’s membrane is a penta-laminar, acellular extracellular matrix located between the RPE and the choriocapillaris.^4^, ^5^, ^6^ Its extracellular matrix is composed primarily of collagens (types I, III, IV, V, and VI) and elastin, along with proteoglycans and glycoproteins, forming a dynamic environment that regulates the bidirectional transport of nutrients such as glucose, amino acids, fatty acids, ions, water, metabolites, and signaling molecules between the retina and choroid.^5^^,^^7^ The permeability and composition of Bruch's membrane change with age and disease, affecting molecular transport.^5^^,^^8^^,^^9^

Bruch’s membrane is relevant for immune regulation by creating a barrier that prevents most complement factors from entering the retina from the choroid, thereby compartmentalizing complement activation and regulation in the eye.^5^^,^^9^^,^^10^ This barrier function is critical for maintaining immune privilege and preventing inappropriate complement-mediated damage to retinal tissues.^10^

The RPE is responsible for the phagocytosis of shed photoreceptor outer segments, a process essential for photoreceptor renewal and retinal homeostasis.^9^ The RPE also secretes a variety of proteins, including matrix metalloproteinases (MMPs), tissue inhibitors of metalloproteinases (TIMPs), epidermal growth factor–containing fibulin-like extracellular matrix protein 1 (EFEMP1), also known as fibulin-3, and complement factors, which modulate extracellular matrix turnover and local immune responses.^8^^,^^10^^,^^11^ These secreted proteins are critical for maintaining the integrity and function of the extracellular matrix and for regulating complement activation in the subretinal space.^8^^,^^10^^,^^11^

Epidermal growth factor–containing fibulin-like extracellular matrix protein 1 is an extracellular matrix protein of which the precise physiological function remains incompletely understood.^12^^,^^13^ Although not a classical extracellular matrix structural protein, EFEMP1 participates in numerous context-dependent protein-protein interactions.^14^ Within the extracellular matrix, EFEMP1 contributes to extracellular matrix homeostasis and structural integrity.^14^, ^15^, ^16^ Epidermal growth factor–containing fibulin-like extracellular matrix protein 1 plays a role in disease pathogenesis, particularly in inherited and AMD.^17^, ^18^, ^19^ The precise expression pattern of EFEMP1 remains a matter of debate. Some studies report that EFEMP1 is not expressed in the RPE or Bruch’s membrane but is restricted to the nerve fiber layer and photoreceptors.^12^ In contrast, a more detailed analysis demonstrated EFEMP1 expression in the RPE as well as in the inner and outer nuclear layers in the retinal periphery. Toward the fovea, expression in the outer plexiform layer increases, while, unexpectedly, expression in the RPE decreases.^16^

Debris from RPE cells, including lipofuscin and other metabolic byproducts, is transported across the extracellular matrix to the choroid via diffusion, while nutrients and oxygen from the choriocapillaris diffuse in the opposite direction to support the RPE and photoreceptors.^5^^,^^6^^,^^9^^,^^10^ The selective permeability of the extracellular matrix is a key determinant of retinal health, and its dysfunction can lead to impaired waste clearance, accumulation of deposits, and progression of retinal diseases.^5^^,^^6^^,^^9^^,^^10^

Extracellular matrix homeostasis is regulated by MMPs and TIMPs, which together control extracellular matrix turnover and remodeling.^20^, ^21^, ^22^ Their exact functions are listed in Tables 1 and 2.

**Table 1:** Function of TIMPs^21^^,^^133^^,^^134^

TIMP	Major Functions	Unique Features	MMP/ADAM Inhibition	Physiological Roles	Pathological Roles
TIMP-1	Inhibition of MMPs, especially MMP-9; regulation of extracellular matrix turnover	Soluble; glycosylated; growth factor-like activity	Broad MMP inhibition, strong for MMP-9	Regulation of cell growth, antiapoptotic; modulation of inflammation; neuroprotection	Elevated in cancer (poor prognosis), fibrosis, chronic inflammation, neurodegeneration
TIMP-2	Inhibition of MMPs, especially MMP-2; regulation of extracellular matrix turnover	Most abundant; interaction with pro-MMP-2	Broad MMP inhibition, strong for MMP-2	Suppression of angiogenesis, modulation of cell-signaling, enhancing of cell adhesion	Tumor suppression, but context-dependent tumor promotion, altered in cardiovascular and fibrotic diseases
TIMP-3	Inhibition of MMPs, ADAMs, ADAMTSs; regulation of extracellular matrix turnover; inhibition of sheddases	ECM-bound; inhibition of ADAMTSs, VEGF; interaction with EFEMP1	Broad inhibition of MMP, ADAM, ADAMTS	Regulation of apoptosis; suppression of angiogenesis; modulation of inflammation	Implicated in tissue fibrosis, cancer (context-dependent), neurodegeneration, cardiovascular disease
TIMP-4	Inhibition of MMPs; regulation of extracellular matrix turnover	Tissue-specific expression; less characterized	Broad inhibition of MMPs, less potent than others	Modulation of cell growth; induction of apoptosis	Altered in cardiovascular disease, cancer progression, tissue remodeling disorders

ADAM = a disintegrin and metalloproteinase; ADAMTS = a disintegrin and metalloproteinase with a thrombospondin type 1 motif; ECM = extracellular matrix; EFEMP1 = epidermal growth factor-containing fibulin-like extracellular matrix protein 1; MMP = matrix metalloproteinase; TIMPs = tissue inhibitor of metalloproteinases.

**Table 2:** Role of MMPs^20^^,^^22^^,^^26^^,^^27^^,^^135^, ^136^, ^137^

MMP	Representative Members	Key Substrates	Major Functions	Physiological Roles	Pathological Roles
All	-	Extracellular matrix proteins, cytokines, growth factors	Degradation of extracellular matrix; cell signaling; cytokine activation	Embryogenesis; morphogenesis; immunity; angiogenesis	Neurodegeneration; cancer; cardiovascular diseases; inflammation; lung diseases, liver diseases, kidney diseases, arthritis
Collagenases	MMP-1, MMP-8, MMP-13	Collagen types I, II, III	Degradation of fibrillar collagens (I, II, III)	Tissue remodeling; bone development; wound healing; cell apoptosis	Arthritis; tumor invasion; inflammation; fibrosis
Gelatinases	MMP-2, MMP-9	Gelatin, elastin, collagen IV	Degradation of denatured collagens, elastin	Angiogenesis; neurogenesis; wound repair; anti-inflammatory; cell apoptosis	Cancer metastasis; inflammation; chronic venous disease; viral infection; arthritis
Stromelysins	MMP-3, MMP-10	Proteoglycans, laminin, fibronectin, elstin	Broad extracellular matrix proteolysis; activation of other MMPs	Wound healing; tissue repair	Cartilage degradation; tumor progression; liver disease; viral infection; lung disease; fibrosis
Matrilysins	MMP-7, MMP-26	Gelatin, casein, fibronectin	Degradation of extracellular matrix proteins; broad substrate specificity	Epithelial cell migration; host defense	Tumor progression; inflammation; lung disease
Membrane-type MMPs	MMP-14, MMP-15, MMP-16	Collagen, gelatin, cell surface proteins	Pericellular proteolysis; activation of MMPs	Cell migration; angiogenesis; morphogenesis; immune response; cell apoptosis	Tumor invasion; tissue destruction
Others	MMP-12, MMP-28	Elastin, other extracellular matrix proteins	Elastase activity; degradation of elastin	Lung tissue remodeling; embryogenesis; tissue remodeling	Emphysema; vascular disease; arthritis; neurological diseases

MMP = matrix metalloproteinase.

Matrix metallopreteinase-2 (gelatinase A) is a key extracellular matrix degrader, particularly in ocular tissues, and is responsible for cleaving type IV and V collagen, laminin, and elastin, often in concert with MMP-9 (gelatinase B).^20^^,^^23^^,^^24^ These enzymes are secreted as inactive pro-MMPs and are activated via proteolytic cleavage or allosteric interactions.^25^, ^26^, ^27^ It is to be clarified whether oxidative stress can activate pro-MMPs.^27^

In healthy individuals, MMPs and TIMPs are present at low levels, maintaining tissue integrity and preventing excessive extracellular matrix degradation.^22^^,^^28^ Dysregulation, with either altered MMP activity or altered TIMP expression, is implicated in pathological conditions such as AMD and other degenerative diseases.^29^, ^30^, ^31^ While MMP-9 is expressed throughout the Bruch’s membrane, MMP-2 is expressed at lower levels in the macular region than in the periphery.^32^

Tissue inhibitor of metalloproteinases-3 (TIMP3) is unique among TIMPs in its ability to bind glycosaminoglycans within the extracellular matrix, which influences its localization and inhibitory function.^21^ This binding property is important for its regulatory role in matrix proteolysis and tissue homeostasis.^21^

Complement factors in a healthy extracellular matrix play a critical role in immune surveillance, homeostasis, and the silent clearance of apoptotic cells and debris, while their activity is tightly regulated to prevent excessive inflammation and tissue damage.^33^, ^34^, ^35^ In the extracellular matrix, complement proteins such as complement factor 3 (C3) and regulatory factors like complement factor H (CFH) bind to matrix components, helping to distinguish between self and nonself and facilitating the removal of altered or damaged host material without triggering a proinflammatory response.^34^, ^35^, ^36^ Complement activation in the extracellular matrix is normally limited by endogenous regulators, including CFH, which binds to extracellular matrix ligands and inhibits the alternative pathway, thereby protecting healthy tissue from complement-mediated injury.^34^^,^^36^

There is insufficient understanding of how extracellular matrix turnover is exactly governed and how it changes with age and with disease.^37^^,^^38^ It is controversially discussed how matrix fragments influence further extracellular matrix remodeling^37^^,^^38^ and inflammation.^2^ There is an ongoing debate on the precise role of extracellular matrix remodeling in ocular diseases including macular degenerations.^7^^,^^8^^,^^39^

## Aging

Extracellular matrix remodeling with aging is characterized by the accumulation and cross-linking of matrix components, driven by oxidative stress and inefficient waste removal.^8^^,^^9^^,^^40^, ^41^, ^42^, ^43^ The accumulation of matrix components such as collagens, elastin, advanced glycation end products, heparan sulfate, and lipid-rich debris leads to a thicker extracellular matrix.^5^^,^^8^^,^^44^ Oxidative stress induces post-translational modifications and fragmentation of extracellular matrix proteins, including collagen and elastin, leading to increased matrix stiffness and impaired cellular interactions.^42^^,^^43^ The photo-oxidative environment of the retina is an optimal source for oxidative modification.^41^ Inefficient waste removal, due to age-related decline in RPE and choriocapillaris function, results in the retention of cellular debris and lipids within Bruch’s membrane.^9^^,^^45^

With age, the hydraulic conductivity reduces.^8^^,^^9^^,^^45^ After reaching a threshold, no effective transport across the extracellular matrix can take place.^8^ This impedes nutrient and oxygen transport and favors the formation of sub-RPE deposits.^8^^,^^9^^,^^45^ The accumulation of advanced glycation end products and cross-linked proteins further stiffens the matrix, exacerbating transport deficits and promoting chronic inflammation.^42^^,^^44^ More cross-linking also impairs the self-cleansing mechanism, by reducing the extracellular matrix’s capability to contract and relax.^8^

Matrix metalloproteinases, especially MMP-2 and MMP-9, which normally degrade extracellular matrix components, show reduced activity with aging, limiting matrix turnover and facilitating abnormal extracellular matrix accumulation.^8^^,^^30^^,^^31^ Active MMPs are entrapped in the extracellular matrix and cannot exert their function properly.^8^ The amount of inactive MMPs increases with age and with thickness of Bruch’s membrane.^46^ Activation is insufficient because pro-MMPs or their activators increasingly bind to the extracellular matrix and get sequestered, removing them from the pool of free pro-MMPs.^8^^,^^32^^,^^47^^,^^48^ Activators of MMPs undergo oxidative changes themselves.^32^ The reduced MMP activity leads to accumulation of matrix components and thickening of the extracellular matrix.^48^ The reduced MMP-2 activity in the macular region might explain why hydraulic conductivity is more reduced, more remodeling happens, and deposits mainly form there.

The active form of TIMP3 is equally bound in the remodeled extracellular matrix. This increases its inhibitory function on MMPs locally and leads to focal accumulations of matrix components and RPE cell debris.^49^

Dysregulation of MMP/TIMP complexes impairs degradation of collagens and elastins, contributing to the persistence of pathological deposits.^8^^,^^30^^,^^40^

With age, EFEMP1 is increasingly expressed.^32^ This causes downregulation of carboxylesterase 1 (CES1), which is important for cholesterol efflux.^14^^,^^50^ Lipids accumulate, contributing to deposit formation.^50^

In age and with accumulating matrix components, high-temperature requirement A serine peptidase 1 (HTRA1) expression is upregulated. High-temperature requirement A serine peptidase 1 cleaves matrix components and EFMP1 and is relevant to maintain tissue integrity.^51^ High-temperature requirement A serine peptidase 1 seems to be expressed less in the macular region than in the periphery.^32^

Rather than being cleared, oxidized accumulations of RPE debris and extracellular matrix proteins persist and trigger a state of low-grade inflammation.^2^ These deposits attract immune cells, which then become trapped within the increasingly thick and rigid extracellular matrix.^2^^,^^11^ As a result, immune efficiency declines and oxidative-stress defense mechanisms weaken.^2^^,^^41^ Complement components—particularly C3 and its fragment complement factor 3b (C3b)—bind to these altered extracellular matrix structures. This interaction activates the alternative complement pathway through a tick-over mechanism.^52^ The aberrant binding of C3 may also hinder its interaction with CFH, reducing complement inhibition and promoting chronic complement activation.^2^^,^^11^

With aging, increased expression of the vitronectin gene leads to greater vitronectin accumulation in the extracellular matrix, further amplifying the persistent low-grade inflammatory environment.^2^

In summary, oxidative stress, inefficient waste removal, and progressive accumulation and cross-linking of extracellular matrix components impair transport across the extracellular matrix, promote sub-RPE deposit formation, and cause low-grade inflammation.^8^^,^^9^^,^^30^^,^^31^^,^^40^, ^41^, ^42^, ^43^, ^44^, ^45^

## AMD

Age-related macular degeneration is the leading cause of irreversible vision loss in the elderly. Although no single causal mutation has been identified, genome-wide association studies have uncovered high-risk variants in several genes involved in extracellular matrix maintenance and immune regulation, including HTRA1,^53^, ^54^, ^55^, ^56^, ^57^ CFH,^57^^,^^58^ TIMP3,^59^^,^^60^ MMP-9 and MMP-2,^29^^,^^30^^,^^59^^,^^60^ C3,^57^^,^^60^^,^^61^ complement factor B (CFB),^62^, ^63^, ^64^ complitment factor I,^65^, ^66^, ^67^ and vitronectin.^65^ Genome-wide association studies meta-analyses have consistently shown enrichment of AMD risk variants in these pathways, and recent studies have expanded the list of associated loci, highlighting the polygenic and multifactorial nature of AMD.^1^^,^^57^^,^^60^^,^^62^^,^^65^^,^^68^, ^69^, ^70^, ^71^

### HTRA1

A major AMD-associated polymorphism lies at the 10q26 locus, affecting HTRA1. Studies diverge regarding its functional consequences. Some reports show that the risk allele leads to HTRA1 overexpression, resulting in aberrant cleavage of EFEMP1 and excessive extracellular matrix fragmentation.^56^^,^^69^^,^^72^ Inefficiently cleaved extracellular matrix proteins may further upregulate HTRA1, creating a self-reinforcing loop of extracellular matrix damage.^56^ Other studies, however, argue that the polymorphism instead reduces HTRA1 expression, suggesting that therapeutic replacement of HTRA1 might restore extracellular matrix homeostasis.^51^^,^^73^ The variant is also associated with downregulation of superoxide dismutase 2, which encodes mitochondrial superoxide dismutase 2—an important antioxidant enzyme—thereby enhancing oxidative stress.^56^^,^^74^

### CFH

High-risk variants in CFH together with HTRA1 account for the greatest heritability in AMD, with both loci exerting major effects on disease risk and progression.^1^^,^^75^ The CFH Y402H polymorphism impairs CFH and its splice variant factor H-like protein 1 (FHL-1) binding to heparan sulfate in the extracellular matrix, reducing local complement inhibition and promoting chronic alternative pathway activation and inflammation.^76^, ^77^, ^78^, ^79^ In AMD, recombinant CFH binds EFEMP1 and age-altered extracellular matrix components with increased affinity, facilitating the formation of EFEMP1-CFH complexes that are abundant in drusen but rare in the retinal periphery.^58^ When CFH is sequestered within the extracellular matrix, its ability to regulate complement activation is diminished, further driving local complement dysregulation and drusen formation.^58^^,^^77^ Notably, wild-type CFH is susceptible to HTRA1-mediated cleavage, whereas AMD-associated mutant CFH resists this process, leading to its accumulation in the extracellular matrix and contributing to defective matrix turnover.^58^

### TIMP3

Age-related macular degeneration‑associated variants in TIMP3 increase its binding affinity for EFEMP1. This may contribute to the formation of a molecular barrier that impedes diffusion across Bruch’s membrane.^80^ In the context of a remodeled or fibrotic extracellular matrix, TIMP3 is sequestered by collagens, resulting in locally elevated concentrations and excessive inhibition of MMPs, thereby reducing extracellular matrix degradation and promoting further matrix accumulation.^8^^,^^30^^,^^31^^,^^40^^,^^81^^,^^82^ This process is implicated in the thickening and decreased permeability of Bruch’s membrane observed in AMD.^8^^,^^40^^,^^82^ Altered TIMP3 also demonstrates increased binding to vitronectin, an inflammation-promoting protein, and to other immunoreactive ligands such as carboxyethylpyrole, albumin, and alpha-1-antitrypsin, further contributing to local immune activation and impaired matrix turnover.^39^^,^^83^^,^^84^ Collectively, these molecular interactions disrupt extracellular matrix homeostasis, facilitate chronic inflammation, and promote the accumulation of pathogenic deposits characteristic of AMD.^8^^,^^30^^,^^31^^,^^39^^,^^40^^,^^80^, ^81^, ^82^, ^83^

### MMPs

Polymorphisms in MMPs, especially in MMP-2 and MMP-9, are associated with increased susceptibility AMD by altering the regulation and activity of these enzymes.^29^^,^^30^ Reduced levels of active MMP-2 and MMP-9 in AMD are a consequence of both genetic variation and increased sequestration of pro-MMPs and high-molecular-weight MMP complexes within the extracellular matrix, which limits their activation and proteolytic function.^30^^,^^48^^,^^85^ Their accumulation with cell blebbing further restricts the pool of active MMPs available for matrix degradation.^4^^,^^85^ In Bruch’s membrane, active MMP-2 is detectable in the periphery but absent within drusen, while inactive forms accumulate in the macular region, contributing to focal impairment of extracellular matrix turnover and the progressive accumulation of matrix components and extracellular deposits.^30^^,^^32^ This regional reduction in proteolytic activity leads to inefficient clearance of debris and lipids, promoting drusen formation and progression of AMD.^30^^,^^85^^,^^86^

### Structural Extracellular Matrix Genes

Alterations in fibulin-5 and fibulin-6 contribute to AMD by disrupting extracellular matrix structure and promoting pathological deposit formation. Mutations in fibulin-5 are associated with abnormal elastogenesis and increased accumulation of basal deposits and drusen beneath the RPE, leading to thickening and reduced permeability of Bruch’s membrane, which impairs nutrient and waste exchange and facilitates disease progression.^87^^,^^88^ Similar changes are observed with fibulin-6, which also participates in extracellular matrix assembly and maintenance.^89^

### Genes Involved in Cholesterol Efflux

Genes such as apolipoprotein E and ATP-binding cassette transporter A1 influence AMD by modulating lipid handling and inflammatory signaling. Apolipoprotein E variants alter the composition and deposition of cholesterol-rich lipoproteins in Bruch’s membrane, increasing the risk of drusen formation and retinal degeneration.^90^^,^^91^ ATP-binding cassette transporter A1 deficiency impairs cholesterol efflux from RPE cells, resulting in intracellular lipid accumulation, local inflammation, and accelerated retinal degeneration.^92^^,^^93^ These changes promote the recruitment and activation of immune cells, further amplifying chronic inflammation and complement activation in the subretinal space.^92^^,^^94^

### Further Complement-associated Genes

High-risk variants in genes such as C3 and CFB, besides CFH, increase susceptibility to AMD by promoting excessive complement activation and chronic inflammation within the retina and choriocapillaris.^63^^,^^64^^,^^95^^,^^96^ Variants in C3 and CFB amplify complement activity, resulting in increased formation of C3b and membrane attack complex, which drive local tissue injury, endothelial cell loss, and photoreceptor degeneration.^63^^,^^64^^,^^96^ Complement factor I inactivates C3b in the presence of cofactors such as CFH, preventing excessive complement activation.^63^^,^^66^^,^^97^ High-risk variants contribute to AMD risk by causing quantitative or qualitative deficiency.^63^^,^^67^^,^^96^, ^97^, ^98^

### EFEMP1

Although no coding EFEMP1 variants are clearly associated with AMD, a copy number variation <1.5 Mb upstream of EFEMP1 has been linked to the disease and may influence regulatory elements controlling EFEMP1 expression.^99^ The EFEMP1 D49A mutation is associated specifically with the cuticular drusen subtype of AMD.^100^

Chronic oxidative stress in aging retinal tissue promotes AMD by inducing protein misfolding and intramolecular cross-linking, which disrupts extracellular matrix integrity and triggers complement activation.^98^^,^^101^ Proteins modified through oxidative stress and altered extracellular matrix structures act as ligands for complement recognition, directly activating the alternative complement pathways and amplifying local inflammation.^95^^,^^102^^,^^103^ Chronic complement activation further damages the RPE and choriocapillaris, exacerbating hypoxia and promoting additional oxidative injury.^95^^,^^104^ The resulting cycle of oxidative stress, extracellular matrix pathology, and complement-mediated inflammation leads to drusen formation and progressive retinal degeneration.^100^, ^101^, ^102^, ^103^

In addition to the effects of genetic variants in complement-associated and extracellular matrix‑associated genes, such as CFH, C3, HTRA1, and TIMP3, chronic oxidative stress acts synergistically to drive AMD pathogenesis by promoting protein misfolding, impaired extracellular matrix degradation, and persistent complement activation.^1^^,^^95^^,^^105^ This multifactorial interplay underlies the progressive nature of AMD and highlights the importance of targeting both oxidative stress and complement pathways in therapeutic strategies.

In early AMD, MMP-2 is overactive—due to aging, stress, and inflammation—and excessively cleaves EFEMP1, leading to defective extracellular matrix remodeling and formation of sticky EFEMP1 fragments that attract complement factors. Abnormal extracellular matrix binds active complement C3, resulting in excessive C3b deposition and chronic activation of the alternative complement pathway, which promotes basal deposit formation and local inflammation in Bruch’s membrane.^11^^,^^106^ Increased MMP-2 activity in this context exacerbates extracellular matrix turnover defects and facilitates the generation of fragments of matrix components and EFEMP1 that serve as damage-associated molecular patterns, further driving complement activation and sterile inflammation.^11^^,^^86^

As patients age, the extracellular matrix becomes stiffer and less permeable.^8^^,^^40^^,^^107^ Despite overexpression, MMP-2 becomes less effective in aged extracellular matrix due to sequestration in high-molecular-weight complexes and reduced activation efficiency, resulting in impaired clearance of EFEMP1 fragments and further drusen buildup.^8^^,^^40^^,^^86^ This age-related decline in MMP-2 activity limits matrix turnover and promotes the accumulation of sticky EFEMP1 fragments and complement factors, perpetuating the cycle of deposit formation and inflammation.^8^^,^^11^^,^^40^

## Doyne Honeycomb Retinal Dystrophy

The p.R345W mutation in the EFEMP1 gene causes Doyne Honeycomb Retinal Dystrophy. It was first described by Robert Doyne in 1899.^108^ The mutation disrupts disulfide-bond formation in the secretory pathway of RPE cells. This mutation impairs proper folding of EFEMP1, resulting in inefficient secretion and retention within the endoplasmic reticulum.^12^^,^^109^^,^^110^ Aberrant accumulation of EFEMP1 leads to drusen formation and later RPE and outer photoreceptor atrophy, the hallmarks of Doyne Honeycomb Retinal Dystrophy.^12^ Epidermal growth factor–containing fibulin-like extracellular matrix protein 1 deficiency results in corneal thinning and increased vascularization.^13^

Deposited EFEMP1 influences extracellular matrix biology through several mechanisms.

1. Unfolded-protein response and VEGF upregulation: Misfolded EFEMP1 triggers endoplasmic reticulum stress and unfolded-protein response, leading to increased VEGF production and promoting choroidal neovascularization.^110^
2. Impaired cholesterol efflux and EGFR pathway inhibition: Mutant EFEMP1 downregulates CES1, reducing cholesterol export and causing lipid droplet accumulation. It also hyperinhibits EGFR signaling, further suppressing CES1 via specificity protein 1 transcription factor downregulation.^50^ Lipids form the bulk of drusen material.^15^
3. Complement activation and C3b deposition: Abnormal extracellular matrix containing mutant EFEMP1 binds C3, activating it through a tick-over process and leading to chronic activation of the alternative complement pathway and excessive C3b deposition, which drives basal deposit and drusen formation.^11^^,^^106^^,^^111^ Complement factor 3 plays a critical role for drusen formation.^14^
4. Interaction with TIMP3 and reduced MMP-2‑dependent extracellular matrix degradation: Epidermal growth factor–containing fibulin-like extracellular matrix protein 1 interacts with TIMP3, and this complex inhibits MMP-2, reducing extracellular matrix turnover and promoting accumulation of matrix components and RPE cell debris.^11^^,^^12^^,^^49^^,^^106^^,^^112^

The accumulation of EFEMP1 reduces transport across the Bruch’s membrane.^113^ The combined effects of chronic inflammation, impaired matrix remodeling, and metabolic stress ultimately drive the transition from drusen to atrophy in Doyne Honeycomb Retinal Dystrophy.^12^

**Figure 1:**
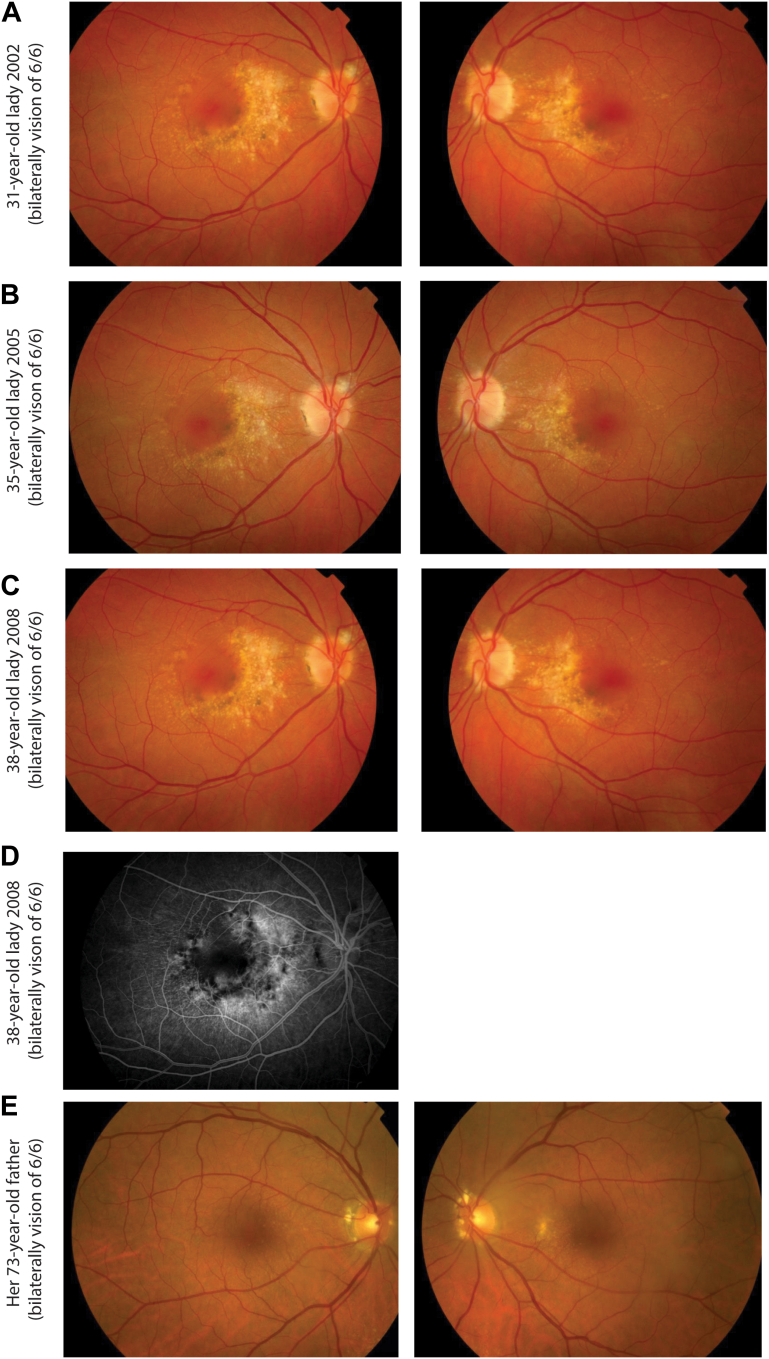
Characteristic retinal imaging illustrating disease evolution in a patient with an EFEMP1 mutation and intrafamilial phenotypic variability, suggesting that factors beyond the EFEMP1 mutation contribute to phenotype development. (**A–C**) Serial fundus photographs obtained over a 6-year follow-up period demonstrate progressive accumulation of deposits surrounding the macula and optic disc. (**D**) Fundus autofluorescence imaging at the most recent follow-up visit reveals a characteristic honeycomb pattern of deposits. (**E**) Fundus photograph of the patient’s father shows a markedly milder phenotype despite his older age. EFEMP1 = epidermal growth factor-containing fibulin-like extracellular matrix protein 1.

The EFEMP1 p.R345W mutation is necessary but not sufficient to determine phenotype severity in Doyne Honeycomb Retinal Dystrophy; additional factors such as age-related oxidative stress, environmental stressors, and lifestyle are required for disease initiation and progression. Substantial intrafamilial and interfamilial variability in clinical presentation and progression is consistently reported, even among individuals with the same EFEMP1 mutation, indicating that modifier factors play a critical role.^114^^,^^115^ Intrafamilial differences and disease progression are exemplarily shown in Figure 1. Drusen formation and disease onset typically occur in mid-adulthood, and progression correlates with age, suggesting that age-related changes in the extracellular matrix, including oxidative stress and chronic inflammation, are important contributors.^12^^,^^114^^,^^115^ Experimental models show that complement activation and immune dysregulation modulate disease severity, further supporting the role of nongenetic factors.^111^^,^^112^^,^^116^^,^^117^ Homozygous and heterozygous carriers of the EFEMP1 mutation show the same phenotype.^118^^,^^119^

In summary, the EFEMP1 mutation alone does not fully determine phenotype severity in Doyne Honeycomb Retinal Dystrophy; age, environmental stressors, and other nongenetic factors are necessary for disease initiation and progression.^112^^,^^114^^,^^115^

## Similarities and Differences

Comparing extracellular matrix changes due to age, the EFEMP1 mutation, and AMD, many similarities in altered pathways appear. However, looking at these changes in more detail, we see that the mechanisms and the speed leading to these alterations differ slightly. This might explain why the phenotypes are similar but not equal.

In all 3 conditions, extracellular matrix is remodeled. This disturbs the extracellular matrix homeostasis, leading to accumulations of RPE cell debris, extracellular matrix proteins, and complement factors. If a threshold of modifications is reached, extracellular matrix homeostasis is dysregulated and drusen start to form. This is shown in Supplementary Figure 1, available at www.ophthalmologyscience.org. In age, remodeling occurs slowly, mainly driven by modifications due to oxidative stress. The changes are small and so are the deposits. The aggregates do not disturb the extracellular matrix balance enough to promote drusen formation. In AMD, high-risk variants of genes coding for proteins involved in extracellular matrix homeostasis and for complement factors occur additionally to the oxidative modifications. Other risk factors such as smoking promote the disease.^104^^,^^107^ The accumulation of these changes causes the threshold to be reached and drusen to form. Given that AMD is a multigenic and multifactorial disease, the combination of modifications that a person can acquire is highly individual. This might explain why there are different subtypes in AMD. In Doyne Honeycomb Retinal Dystrophy, the EFEMP1 mutation is the major predisposing factor for drusen formation. It disturbs the extracellular matrix balance almost enough to cause drusen formation on its own. However, given that there are interocular, intrafamilial, and interfamilial differences,^118^^,^^119^ some additional modifications, due to oxidative stress or aging, are necessary for drusen formation. As the EFFEMP1 mutation causes almost enough disturbance to reach the threshold, there is little space for different further stress accumulation and the phenotype looks very homogenous among Doyne Honeycomb Retinal Dystrophy patients.

While these changes occur over many decades in AMD,^104^ the EFEMP1 mutation makes the disease progress much faster. Deposits start to form from childhood or early adulthood.^115^ The EFEMP1 mutation might thus be a critical hazard for drusen formation. It might speed up disease development by several decades. This different speed could also explain differences in phenotypes as the environment has more or less time to adapt.

In all cases, extracellular matrix gets thicker and stiffer. Accumulation of matrix components occurs because less degradation and more cross-linking of collagens happen and the transport of matrix components across the extracellular matrix from RPE to the choroid is impaired.^8^^,^^40^ Inactive MMPs get sequestered in the remodeled extracellular matrix, entrapped active MMPs lose their proteolytic potential, and entrapped active TIMPs locally increase their inhibiting function on MMPs, resulting in reduced degradation of extracellular matrix components.^48^^,^^86^ The hydraulic conductivity decreases, impairing transport across the extracellular matrix.^8^ In AMD, these changes are exacerbated with high-risk variants of MMPs, TIMP3, and structural matrix proteins as well as oxidative changes. In Doyne Honeycomb Retinal Dystrophy, abnormally folded EFEMP1 accumulates and directly disturbs extracellular matrix balance and turnover and transport across the extracellular matrix.^113^^,^^120^

Epidermal growth factor–containing fibulin-like extracellular matrix protein 1 is overexpressed in age. While it accumulates in the periphery, no expression is detected in the central retina.^16^ It is secreted and degraded efficiently and does not accumulate there. Only in disease conditions does it accumulate in the macular region.^80^^,^^113^^,^^121^ In AMD, it accumulates beneath the RPE and above drusen. In Doyne Honeycomb Retinal Dystrophy, it is secreted inefficiently and accumulates within RPE cells, beneath them, and above drusen. In both conditions, EFEMP1 is not a major component of drusen themselves.^12^ While reduced degradation through alterations in HTRA1 can cause EFEMP1 accumulation in AMD,^56^ the R345W EFEMP1 mutation is the causer in Doyne Honeycomb Retinal Dystrophy.^112^ Given this difference between healthy aging and disease, submacular EFEMP1 accumulation and their increased binding to TIMP3 might be critical for igniting the drusen accumulation.

High-temperature requirement A serine peptidase 1 is expressed in healthy aging as well as macular degenerations. It degrades EFEMP1 additionally to other matrix proteins seen in aging.^56^ The sticky fragments it forms attract complement factors.^56^ They might be relevant for drusen formation and chronic activation of the alternative complement pathway. High-temperature requirement A serine peptidase 1 downregulates CES1, which impairs cholesterol efflux.^50^ This promotes lipid accumulations. Altered HTRA1 cleaves less EFEMP1, leading to more EFEMP1 accumulation.^56^

The remodeled and fragmented extracellular matrix attracts complement factors and lipids. Cellular debris, oxidized lipids, and abnormal extracellular matrix proteins act as danger-associated molecular patterns. Complement factor 3 binds to altered extracellular matrix structures and is being activated through a tick-over process.^11^ This activates the alternative pathway of the complement system.^122^ Activation of the alternative complement pathway occurs at low levels and in a regulated way in healthy aging. Women show reduced levels of complement activation compared to men.^123^ The complement activation is dysregulated in disease.^1^ In AMD, a high-risk variant of CFH reduces the inhibition of complement activation, leading to chronic overactivation of the alternative pathway.^89^^,^^124^ Altered CFH binds EFEMP1 with higher affinity than does wild-type CFH.^58^ In Doyne Honeycomb Retinal Dystrophy, C3 and CFB are upregulated.^111^^,^^116^ The mutated EFEMP1 binds more easily to C3 than wild-type EFEMP1.^11^

**Figure 2:**
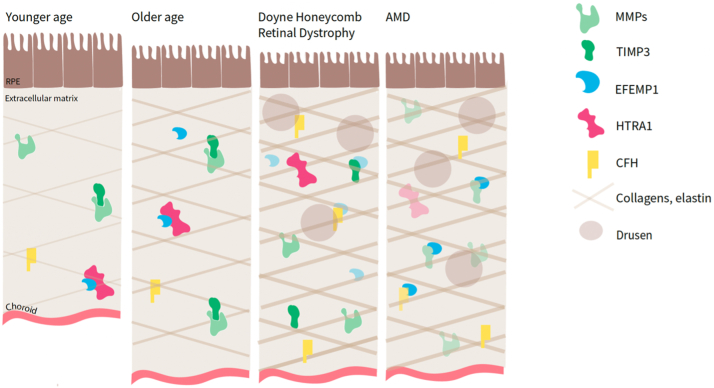
Schematic representation of extracellular matrix protein interactions in aging and disease, illustrating major components that are altered under pathological conditions. This figure illustrates age-associated and disease-associated thickening and increased cross-linking of the extracellular matrix, resulting in altered matrix function. The schematic highlights the entrapment of matrix-degrading enzymes and extracellular matrix proteins within the modified matrix. Lighter-colored particles represent structural alterations arising from genetic variation or oxidative modification, and the figure emphasizes preferential binding among altered components compared with unmodified counterparts. The relative thicknesses of the retinal pigment epithelium (RPE), extracellular matrix, and choroid are intentionally not drawn to scale. AMD = age-related macular degeneration; CFH = complement factor H; EFEMP1 = epidermal growth factor-containing fibulin-like extracellular matrix protein 1; HTRA1 = high-temperature requirement A serine peptidase 1; MMP = matrix metalloproteinase; TIMP = tissue inhibitors of metalloproteinases.

This interaction of RPE cell debris, fragments of extracellular matrix proteins, and complement factors drives drusen formation.^52^^,^^125^ These changes and interactions are visualized in Figure 2 and are depicted in more detail in Supplementary Figure 2, available at www.ophthalmologyscience.org.

**Figure 3:**
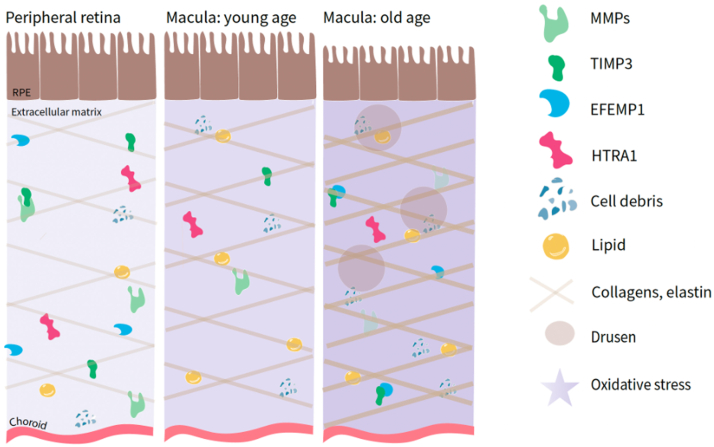
Schematic overview of regional differences in extracellular matrix homeostasis between the peripheral and macular retina. This figure illustrates regional differences in the interactions of key extracellular matrix proteins between the peripheral and macular retina. Lighter-colored particles represent structural alterations arising from genetic variation or oxidative modification. The relative thicknesses of the retinal pigment epithelium (RPE), extracellular matrix, and choroid are intentionally not drawn to scale. EFEMP1 = epidermal growth factor-containing fibulin-like extracellular matrix protein 1; HTRA1 = high-temperature requirement A serine peptidase 1; MMP = matrix metalloproteinase; TIMP = tissue inhibitors of metalloproteinases.

Drusen might form in the macular region but not equally in the peripheral retina as there is more accumulation of extracellular matrix components and debris in the submacular region of Bruch's membrane compared to other regions because the submacular Bruch's membrane has a higher concentration of noncollagenous proteins and protein-containing debris, increased lipid deposition, and greater age-related thickening. As illustrated in Figure 3, together, this promotes trapping of macromolecules and impairs matrix turnover.^126^ This regional difference is attributed to the unique metabolic demands and high density of photoreceptors and RPE in the macula, leading to increased production and deposition of metabolic byproducts and extracellular matrix components.^107^^,^^126^ Additionally, the submacular Bruch's membrane undergoes more pronounced age-related changes, such as increased cross-linking of collagen, accumulation of advanced glycation end products, and alterations in glycosaminoglycan composition, all of which reduce its permeability and facilitate the retention of debris and matrix material.^5^^,^^126^ The nonhomogenous structure and transport could lead to focal entrapment.^5^ Epidermal growth factor–containing fibulin-like extracellular matrix protein 1 increasingly accumulates in the macular region, where it is normally absent.^17^ Epidermal growth factor–containing fibulin-like extracellular matrix protein 1 binds to TIMP3 there, enhancing the physical barrier in the extracellular matrix.^80^ Impaired MMP activity with aging, especially in the macular region,^32^ further limits the clearance of these deposits, exacerbating their accumulation in the macular region.^8^^,^^9^ High-temperature requirement A serine peptidase 1, the other cleaving enzyme of EFEMP1, is expressed at lower levels in the macular region.^16^

This review does not look at components of drusen in detail. Further analysis of molecular changes from drusen development to outer photoreceptor and RPE atrophy is warranted. Finally, we did compare mechanisms leading to choroidal neovascularisation in different disease conditions. These aspects could help us refine differences and similarities between AMD and Doyne Honeycomb Retinal Dystrophy.

## Therapeutical Potential

While no therapy targeting extracellular matrix changes has been approved either for AMD or Doyne Honeycomb Retinal Dystrophy in the United Kingdom, several clinical or preclinical studies explore interventions.

Two clinical studies target complement inhibition, with a C3 respectively complement factor 5 inhibitor.^127^, ^128^, ^129^ Antisense-oligonucleotides, antibodies, and small-molecules targeting CFB are being investigated, part of them in clinical trials.^130^

One study uses laser treatment to induce small lesions in the RPE cells. When other RPE cells move to the lesion site to repair the lesions, they release active MMPs.^131^

In another study, triterpenoid saponins have been supplemented. They showed that saponins improve transport across the Bruch’s membrane and the recovery of photoreceptor sensitivity. This might halt or reverse AMD progression.^132^

Different studies have tried to inhibit MMPs or TIMPs. They were not fully successful, which is not completely surprising given that MMPs and TIMPs might have a protective effect on the extracellular matrix.^30^ On contrary, in vitro studies have shown that RPE-specific MMP-2 supplementation, receptor for advanced glycation end-products-antagonistic peptide, and a small molecule inhibitor of secreted phospholipase A2 group IIA can reduce drusen formation in AMD.^86^

Epidermal growth factor–containing fibulin-like extracellular matrix protein 1 has been targeted with antisense oligonucleotides for patient-derived RPE cells with the EFEMP1 mutation, showing a reduction in extracellular matrix deposits.^15^

No clinical study has so far been conducted on Doyne Honeycomb Retinal Dystrophy. Due to similarities between the disease entities, the drugs tested on AMD might be tested on Doyne Honeycomb Retinal Dystrophy. Further studying the changes in the extracellular matrix helps us identify additional therapeutical targets.

## Conclusion

Alterations in the extracellular matrix in patients suffering from Doyne Honeycomb Retinal Dystrophy respectively AMD are similar. While the EFEMP1 mutation is the leading driver in Doyne Honeycomb Retinal Dystrophy, oxidative stress due to aging and risk-increasing genetic modifications drive pathogenesis in AMD. Thereby, the interaction of EFEMP1 and TIMP3 might be a crucial point in drusen formation. We hypothesize that the pathogenesis in both diseases is similar but the speed leading to the development of the phenotype differs. While changes in AMD occur over most of the person’s life, they are packed into 30 to 40 life-years in a person with EFEMP1 mutation. This mutation can be seen as an enormous hazard for disturbing the extracellular matrix homeostasis and causing drusen development, while in AMD many little insults are necessary to achieve the same amount of disbalance. Age is one such hazard, which on its own is not sufficient to cause drusen formation but only leads to deposits and low-grade inflammation.

To understand the similarities and differences between AMD and Doyne Honeycomb Retinal Dystrophy better, one warrants studying extracellular matrix proteins in more detail. It is necessary to look at their expression with age and under different stress conditions. So far, we do not know whether sex or vascular changes influences phenotype progression in Doyne Honeycomb Retinal Dystrophy. This needs to be studied.

We suggest further using Doyne Honeycomb Retinal Dystrophy as a disease model for AMD. Studying the monogenetic disease might help us find therapeutical targets for AMD.

## Declaration of Generative AI and AI-Assisted Technologies in the Writing Process

During the preparation of this work the authors used ChatGPT (OpenAI, Version 5) and Open Evidence in order to assist with literature exploration, synthesis, and language refinement. After using this tool/service, the authors reviewed and edited the content as needed and take full responsibility for the content of the publication.
