# Supplementary material for: Interactions Between Epidermal Growth Factor–Containing Fibulin-Like Extracellular Matrix Protein 1 and Tissue Inhibitor of Metalloproteinases-3 and Relevance to Age-Related Macular Degeneration
Source: Ophthalmol Sci. 2026 Jun 16;6(8):101287. doi: 10.1016/j.xops.2026.101287 (PMC13449369; doi:10.1016/j.xops.2026.101287)
Supplement: Supplementary file 1 [file mmc1.pdf]

Supplementary Figure 1. Factors contributing to extracellular matrix dysfunction and the subsequent development of drusen.

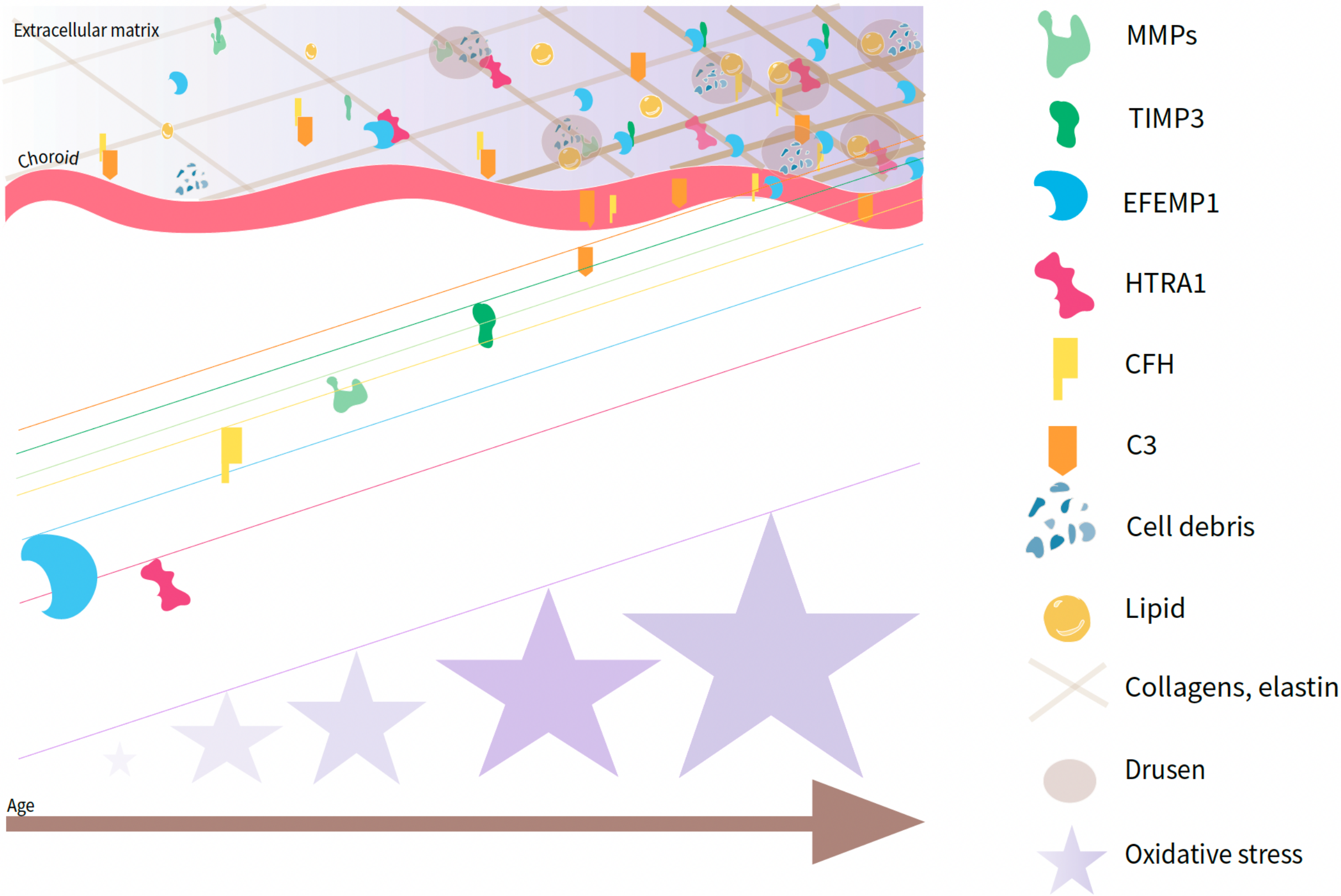

This illustration depicts the age-associated increase in oxidative stress and its contribution to drusen formation. High-risk variants of extracellular matrix proteins or complement factors, arising from genetic variation or oxidative modification, are shown to elevate susceptibility to drusen development. The EFEMP1 mutation is highlighted as a major risk factor that, while substantial, is insufficient on its own to induce drusen formation. The figure emphasizes that drusen develop once multiple oxidative and/or genetic alterations accumulate and collectively surpass a pathogenic threshold. The relative thicknesses of the extracellular matrix and choroid are intentionally not drawn to scale.
