# Supplementary material for: Interactions Between Epidermal Growth Factor–Containing Fibulin-Like Extracellular Matrix Protein 1 and Tissue Inhibitor of Metalloproteinases-3 and Relevance to Age-Related Macular Degeneration
Source: Ophthalmol Sci. 2026 Jun 16;6(8):101287. doi: 10.1016/j.xops.2026.101287 (PMC13449369; doi:10.1016/j.xops.2026.101287)
Supplement: Supplementary file 2 [file mmc2.pdf]

*Supplementary Figure 2. Extracellular matrix alterations associated with aging, Doyne honeycomb retinal dystrophy, and age-related macular degeneration.*

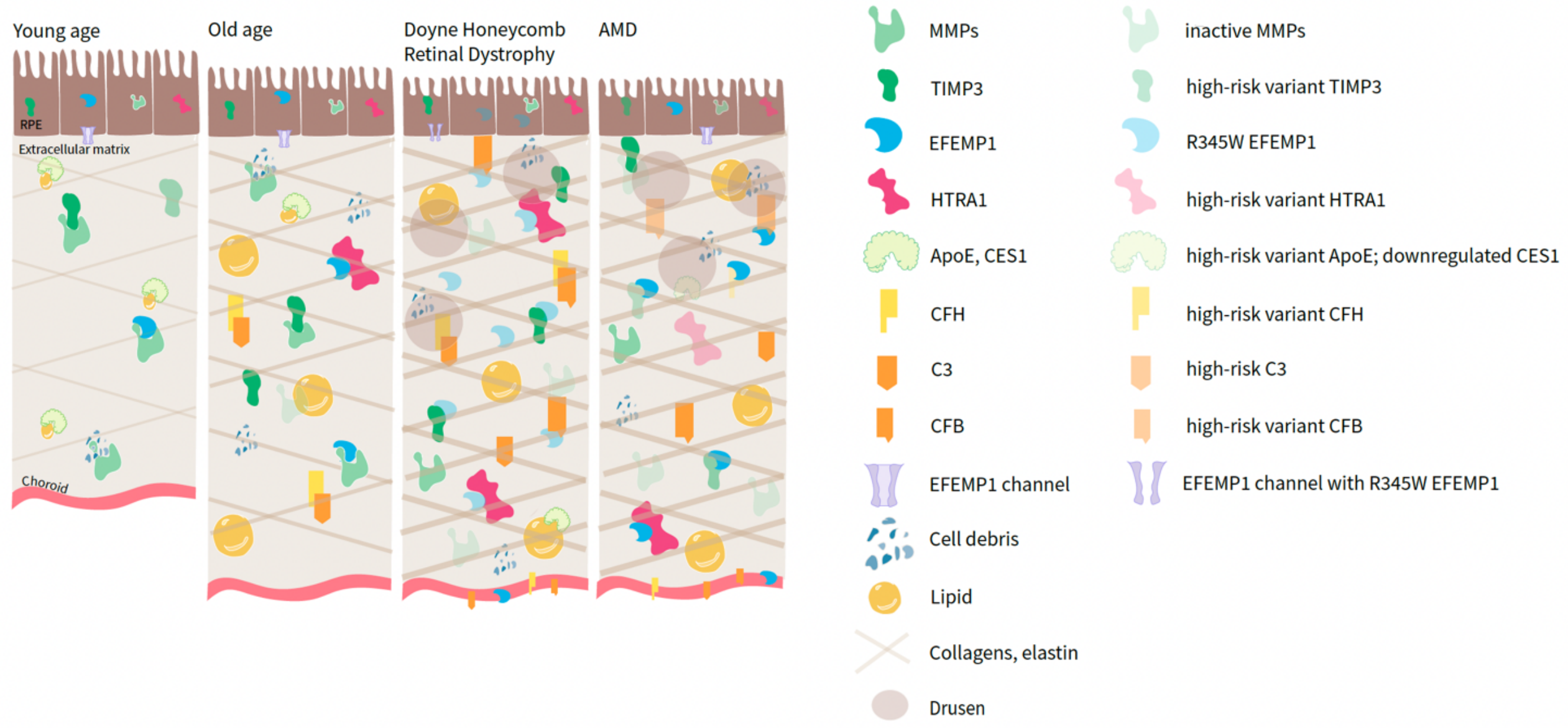


This figure provides a detailed view of extracellular matrix alterations and illustrates how progressive modification of matrix components contributes to drusen formation. The relative thicknesses of the retinal pigment epithelium (RPE), extracellular matrix, and choroid are intentionally not drawn to scale.
